# Optimization of Hot Embossing Condition Using Taguchi Method and Evaluation of Microchannels for Flexible On-Chip Proton-Exchange Membrane Fuel Cell

**DOI:** 10.3390/mi15081033

**Published:** 2024-08-14

**Authors:** Yubo Huang, Han Gao, Zhiheng Wu, Hongyang Xiao, Cao Xia, Yuanlin Xia, Zhuqing Wang

**Affiliations:** 1 School of Mechanical Engineering, Sichuan University, Chengdu 610065, China; huangyubo@scu.edu.cn (Y.H.); 13903101160@163.com (H.G.); xiacao_30@scu.edu.cn (C.X.); yuanlin.xia@scu.edu.cn (Y.X.); 2 Pittsburgh Institute, Sichuan University, Chengdu 610225, China; wuzhiheng@aactechnologies.com (Z.W.); 13990184114@163.com (H.X.)

**Keywords:** PEMFC, hot embossing, microchannels, the Taguchi method

## Abstract

Hot embossing is a manufacturing technique used to create microchannels on polymer substrates. In recent years, microchannel fabrication technology based on hot embossing has attracted considerable attention due to its convenience and low cost. A new evaluation method of microchannels, as well as an approach to obtaining optimal hot embossing conditions based on the Taguchi method, is proposed in this paper to fabricate precise microchannels for a flexible proton-exchange membrane fuel cell (PEMFC). Our self-made hot embossing system can be used to fabricate assorted types of micro-channel structures on polymer substrates according to various applications, whose bottom width, top width, height and cross-sectional area vary in the aims of different situations. In order to obtain a high effective filling ratio, a new evaluation method is presented based on the four parameters of channel structures, and the Taguchi method is utilized to arrange three main factors (temperature, force and time) affecting the hot embossing in orthogonal arrays, quickly finding the optimal condition for the embossing process. The evaluation method for microchannels proposed in this paper, compared to traditional evaluation methods, incorporates the area factor, providing a more comprehensive assessment of the fabrication completeness of the microchannels. Additionally, it allows for the quick and simple identification of optimal conditions. The experimental results indicate that after determining the optimal embossing temperature, pressure and time using the Taguchi method, the effective filling rate remains above 95%, thereby enhancing the power density. Through variance analysis, it was found that temperature is the most significant factor affecting the hot embossing of microchannels. The high filling rate makes the process suitable for PEMFCs. The results demonstrate that under optimized process conditions, a self-made hot embossing system can effectively fabricate columnar structure microchannels for PEMFCs.

## 1. Introduction

In recent years, proton-exchange membrane fuel cell (PEMFCs) have been attracting much attention on account of their advantages of high energy conversion, their outstanding reliability and their non-pollution [1,2,3,4,5,6,7,8,9,10,11,12]. However, PEMFC technology still faces many challenges in practical applications, such as high cost, short service life and low power density [13,14,15,16,17,18,19]. Jie Jin et al. proposed that a certain amount of oxygen applied to TiN coating could reduce the potential effect during vehicle startup and shutdown, so that the service life of the PEMFC can be increased [20]. In addition, the structure of a fuel cell can be designed and modified to improve its performance. Tominaka. S. et al. designed a new on-chip fuel cell and then improved its performance through numerical simulation [21,22], and the proposal of an on-chip fuel cell provides a new idea for making PEMFCs.

Since both the design and fabrication of microchannel structures on the surface of flexible polymer substrates are exceedingly essential for the application of PEMFCs, we have been showing great solicitude for developing high-performance on-chip fuel cells. We have proposed an inkjet printing method for the deposition of Nafion ionomers, and the results show that the preparation method of cathode electrode affects the performance of the PEMFC [23,24,25,26,27,28]. And a pillared fuel cell’s structure on flexible polymers, which transmit protons through microchannels, has been proposed by our research group [29]. In this case, the approach to fabricate microchannels becomes a prominent aspect of improving the performance of flexible fuel cells. Traditional PEMFCs have a low efficiency and large size, and the efficiency of hot embossing equipment is also low. Therefore, we propose a new evaluation method for microchannels, which takes into account the impact of the cross-sectional area of the microchannels and provides a comprehensive assessment. Table 1 shows a comparison of different evaluation methods. Additionally, the Taguchi method is used to quickly identify the optimal conditions and the influence of each factor on the quality of membrane formation. Micro-hot-embossing technology, as a widely investigated precise machining method [30], can realize the embossing of microchannel structures by using a mold. Manufacturing microchannels through hot embossing technology helps reduce costs and improve filling ratio in an effort to promote the performance of fuel cells (e.g., increase their power density) [31,32,33,34,35,36]. Therefore, a self-made hot embossing system is developed to fabricate microchannel structures on flexible polymers.

**Figure 1:**
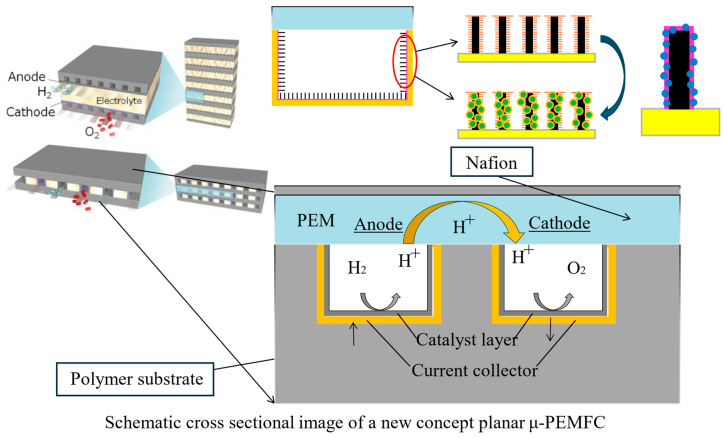
Schematic of a new concept planar micro-PEMFC.

The new on-chip fuel cell designed by our research group is shown in Figure 1. The micro-carbon tube is deposited on the surface of fabricated microchannels to increase the power density of the PEMFC system. The PEMFCs utilize a proton-exchange membrane to directly convert hydrogen and oxygen into electrical energy. At the anode, hydrogen molecules split into protons and electrons. The protons migrate through the proton-exchange membrane to the cathode, while the electrons flow through an external circuit, generating an electric current. At the cathode, oxygen combines with the protons and electrons to form water. The parallel PEMFC developed by us can improve its efficiency through stacking, while significantly reducing the overall size. The self-made hot embossing system is used to fabricate microchannels on the polymer to obtain an improved filling ratio. The system shows excellent stability in application and facilitates the current collection of on-chip fuel cells. In general, as a precise machining technology, the manufacturing accuracy of hot embossing is influenced by certain factors.

In this study, our research group mainly explores the effect of embossing time, embossing temperature and embossing force on polymer filling ratio, and then achieves the optimal conditions. A new evaluation method aiming at the filling ratio of microchannels is proposed. Then, using the Taguchi method, different values of the three hot embossing factors are served as experimental conditions to obtain the optimal condition. As a reliable and efficient experimental statistical method, the Taguchi method helps minimize the number of tests and leads to easy data analysis [41,42,43,44,45,46]. With the help of optimal fabricating conditions, the new on-chip fuel cell can be used in many fields with an increased power density, such as micro-sensors, microchips and so on.

## 2. Equipment Condition and Fabrication Method

### 2.1. Self-Made Hot Embossing Equipment

**Figure 2:**
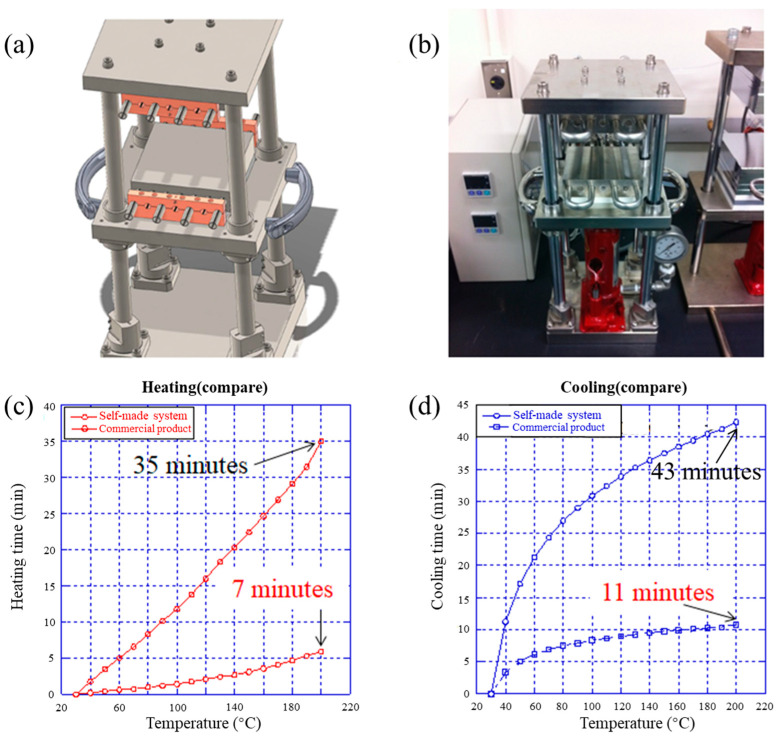
The self-made micro-hot-embossing system and the self-made hot embossing equipment in comparison with the commercial product: (**a**) the mold of the self-made hot embossing equipment; (**b**) a physical drawing of the self-made hot embossing equipment; (**c**) a comparison of the heating times between the self-made hot embossing equipment and a commercial product; (**d**) a comparison of the cooling times between the self-made hot embossing equipment and a commercial product.

To make high-quality microchannels on polymer substrates, high-efficiency hot embossing equipment is extremely crucial. Our self-made hot embossing system is shown in Figure 2a,b, with more details shown in Figure S1 (Supplementary Materials). Hot embossing at the work space is operated in the middle of the chamber. The heating process is achieved through thermocouples, which are inserted into the lower and upper stages, respectively, and the surface temperature of the device reaches a certain value. Meanwhile, embossing force is generated through the force controller below the work space, which can maintain a stable and continuous pressure output. Figure 2c,d shows the self-made hot embossing equipment in comparison with the commercial product. Figure 2c,d shows the performance of common products with heating time and cooling time. The equipment from the company used 35 min to heat the stage to 190 °C and 43 min to cool the stage to room temperature. In comparison, the performance of a self-made hot embossing machine for heating is shown in Figure 2c, which only takes 7 min to heat the stage to 190 °C. The performance of cooling shows that it takes 11 min to cool the stage to room temperature. The results show that our self-made hot embossing machine is a great method to fabricate high-quality microchannels for our research, with a better performance in heating and cooling than common products.

### 2.2. Hot Embossing Method and Conditions

**Figure 3:**
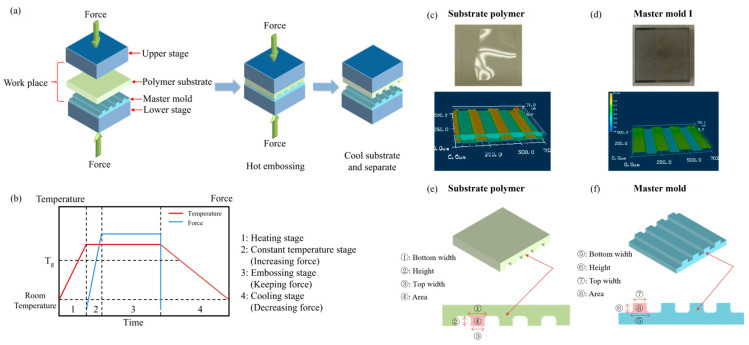
The processes of hot embossing and the substrate polymer and master mold used in hot embossing processes and the cross-section structures of the master mold and the substrate polymer: (**a**) the processes of hot embossing; (**b**) the temperature and force acting stages in the hot embossing process; (**c**) the structures of the substrate polymer before and after the embossing process; (**d**) the structures of the master mold before and after the embossing process; (**e**) the cross-section structures of the substrate polymer; and (**f**) the cross-section structures of the master mold.

There are three processes involved in hot embossing, as shown in Figure 3a. We completed the experiment with our self-made hot embossing equipment. First, the master mold and the polymer substrate are put into the work space, which is in the middle of the chamber, and the heating stage is started. Then, the mold and the substrate are embossed for a period to fabricate microchannels. Finally, the mold and the substrate are separated after the cooling stage. At this time, the microchannels can be observed forming on the polymer substrate. The curves of temperature and force during the hot embossing process are plotted in Figure 3b. The work space is heated and reaches a predetermined temperature before embossing. After that, force is applied and gradually increases for a period of time. Then, both the temperature and force remain constant in the embossing stage. Eventually, the work space is cooled and depressurized before separating the mold and the substrate. In this case, we can summarize that there are four stages for temperature and three stages for force, as shown in Figure 3b.

There are three main factors affecting the quality of microchannels, namely, embossing temperature, embossing force and embossing time, which play a significant role during the hot embossing process.

### 2.3. Material

The structures of the substrate polymer and the master mold before and after the embossing process are shown in Figure 3c and Figure 3d, respectively. We used polystyrene (PS) polymer as the substrate material to complete follow-up experiments. In order to fabricate microchannels, the embossing temperature should be greater than the glass transition temperature, which is 100 °C for PS polymer. The size of the master mold (Mold I) was approximately 97.85 μm for the top width, 127.17 μm for the bottom width, 20.65 μm for the depth and 2216 μm^2^ for the cross-sectional area.

In our experiments, both the substrate polymer and he master mold were placed in the work space. The conditions for maximizing effective filling ratios were obtained by changing experimental condition factors, including the embossing temperature, embossing force and embossing time.

### 2.4. Evaluation Method of Fabricated Microchannels

The cross-section structures of the master mold and the substrate polymer are shown in Figure 3e,f. A laser microscope was used to observe and measure the PS polymer structures after embossing. The top width, bottom width, height and cross-sectional area of the microchannels are the four parameters involved in evaluating the filling rate. Therefore, four effective filling ratios are defined to evaluate the filling rate, including the top width ratio (P_T_), the bottom width ratio (P_B_), the area ratio (P_A_) and the height ratio (P_H_), and their definitions are given below:

(1) $$P_{T}=\frac{Polymer\;top\;width}{Mold\;bottom\;width}\times 100\%$$

(2) $$P_{B}\;=\frac{Polymer\;bottom\;width}{Mold\;top\;width}\;\times \;100\%\;(W_{bottom}^{plymer}\;\leq \;W_{top}^{mold})$$

(3) $$P_{B}=\frac{Mold\;top\;width}{Polymer\;bottom\;width}\;\times \;100\%\;(W_{bottom}^{plymer}\;>\;W_{top}^{mold})$$

(4) $$P_{A}=\frac{Polymer\;area}{Mold\;area}\times 100\%$$

(5) $$P_{H}=\frac{Polymer\;height}{Mold\;height}\times 100\%$$

It should be noted that when the bottom width of the polymer is greater than the top width of the mold, P_B_ is defined as the top width of the mold divided by the bottom width of the polymer. Otherwise, P_B_ is defined as the bottom width of the polymer divided by the top width of the mold, as shown in Equations (2) and (3). Under ideal optimal conditions, the values of all four effective filling ratios approach 1. However, these four filling ratios cannot reach up to 1 in practical experiments. Our experiments aimed to maximize the effective filling ratios through adjusting the experimental conditions.

## 3. Experimental Results

### 3.1. Factor Design

**Figure 4:**
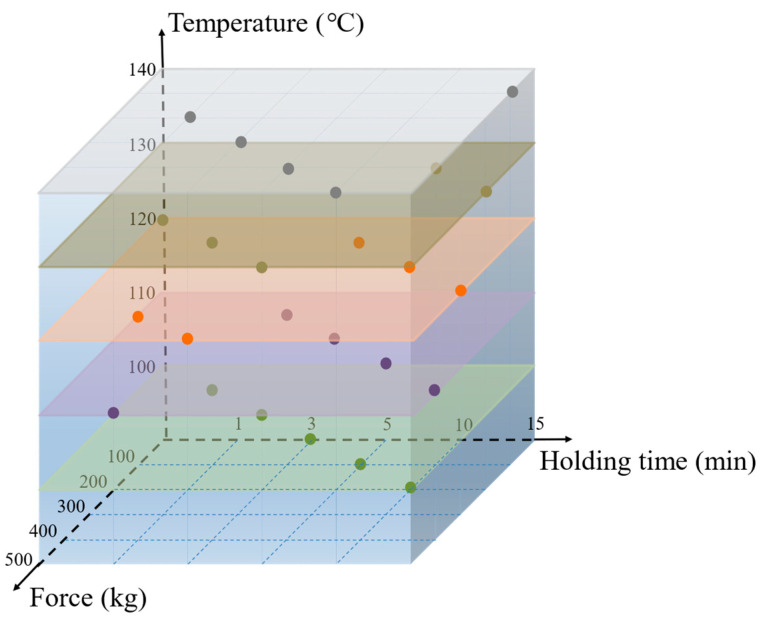
Three main factors arranged by orthogonal arrays using the Taguchi method.

As mentioned before, there are three main factors affecting the filling ratios of the micro-channel structures, namely, the embossing temperature, the embossing force and the embossing time. We divided these factor values into five levels in advance, as shown in Table 2 and Figure 4. For example, the first level of embossing temperature, embossing force and embossing time is configured to 100 °C, 100 kg and 1 min, respectively. Each level of the factors is arranged and combined sequentially as experimental conditions, and the Taguchi method is utilized in the experiments to analyze and quickly obtain optimal embossing conditions.

### 3.2. Experimental Results and Mean Value Analysis Method

The experimental results are shown in Table 3, and the last column represents the average value of the four types of filling ratios. Based on the Taguchi method, orthogonal arrays are formed through combining different levels of embossing temperature, embossing force and embossing time, and the corresponding filling ratios are computed, as shown in Table 3.

In addition, we calculated the mean values of the average filling ratios for the three main factors at each level separately; $K_{i}^{f}$ represents the mean value of the average filling ratios for a specific factor (e.g., embossing temperature) of a specific level (e.g., 100 °C, level 1, *i* = 1) combined with other two factors. For example, $K_{1}^{temp}$ represents the mean value of the average filling ratios at the embossing temperature of 100 °C in the experiment, which can be calculated as follows:

(6) $$K_{1}^{temp}=\frac{90.73+94.57+95.00+95.38+97.63}{5}=94.66$$

The results of the mean value of the average filling ratio for three factors are shown in Table 4. The last column of Table 4 shows the range of the filling ratio, denoted as $R^{f}={\left(K_{i}^{f}\right)}_{\max}-{\left(K_{i}^{f}\right)}_{\min}$. For instance, $R^{temp}$ = 98.20 − 94.66 = 3.54.

### 3.3. Optimum Temperature Analysis

**Figure 5:**
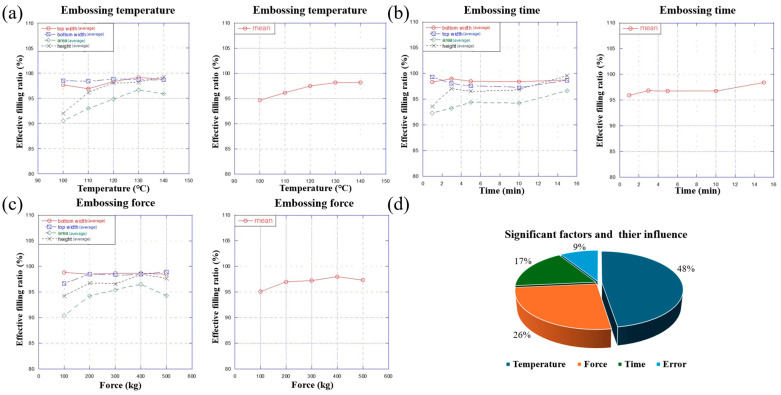
Effective filling ratio for different levels of embossing temperature, embossing force and embossing time, and the proportion of the influence of the factors and error on the experimental results: (**a**) effective filling ratio for different levels of embossing temperature; (**b**) effective filling ratio for different levels of embossing time; (**c**) effective filling ratio for different levels of embossing force; and (**d**) the proportion of the influence of the factors and errors on the experimental results.

Through the analysis of the means, we can determine the optimum conditions for embossing PS polymer. As previously mentioned, the embossing temperature needs to be set above the glass transition temperature. Therefore, the embossing temperature range in the experiment is from 100 °C to 140 °C. After repeating the experiment three times, we recorded the results and calculated the mean values of the average filling ratio ($K_{i}^{temp}$), as shown in Figure 5. Figure 5a shows that the value of $K_{i}^{temp}$ increases from 100 °C to 130 °C and then decreases when it exceeds 130 °C. It can be concluded that the optimum embossing temperature for PS polymer is 130 °C among the five levels tested. It is also noteworthy that the effective filling ratio versus various parameters in the microchannel fabrication process, as shown in Figure 5a, indicates that the filling ratios for the top width, bottom width and height are consistently near 98% across the temperature range of 100 °C to 140 °C, demonstrating the stable and precise control of these dimensions throughout the process. However, the area parameter shows more variation, starting at around 90% at lower temperatures and stabilizing near 98% as the temperature approaches 130 °C. This suggests that higher embossing temperatures enhance the filling efficiency for the area parameter, while other dimensions are well-controlled throughout the process.

### 3.4. Optimum Force Analysis

Accordingly, as shown in Figure 5, the optimal force condition is determined through the analysis of the means. In this experiment, the embossing force ranges from 100 Pa to 500 Pa. After repeating the experiment three times, we recorded the results and calculated the mean value of the average filling ratios ($K_{i}^{force})$. Figure 5b shows that the value of $K_{i}^{force}$ varies zigzaggingly from 100 Pa to 500 Pa. Basically, it tends to rise from 100 Pa to 400 Pa and fall when the force is greater than 400 Pa. This brings us to the conclusion that the optimum embossing force for PS polymer is 400 Pa among the five levels.

### 3.5. Optimum Time Analysis

The optimum time for embossing PS polymer is obtained similarly, as shown in Figure 5. In this experiment, the embossing time ranges from 1 min to 15 min. After repeating the experiment three times, we recorded the results and calculated the mean value of the average filling ratios ($K_{i}^{time})$. As can be seen in Figure 5c, the value of $K_{i}^{time}$ increases slowly from 1 min to 15 min and finally reaches the maximum value at 15 min. It can be concluded that the optimum embossing time for PS polymer is 15 min among the five levels.

Based on the optimum values of embossing temperature, force and time, we can draw the conclusion that the optimal effect can be achieved under the experimental conditions of 130 °C, 400 Pa and 15 min. On the contrary, under the condition of low temperature and low force for a short time, the embossing effect is not ideal, which also conforms to the expectation at the initial stage of the experimental setting. Obviously, embossing between the mold and the polymer is not sufficient under the condition of low temperature and low force for a short time.

### 3.6. Analysis of Variances (ANOVA)

An analysis of variances (ANOVA) is conducted in this section, which is used to calculate the influence degree of each factor on the experimental results. Firstly, determine the degree of freedom for the three influential factors and the error, which are given by the following formulas:

(7) $$DOF^{f}=N_{level}^{f}-1$$

(8) $$DOF^{total}=n-1\;\;$$

(9) $$DOF^{error}=DOF^{total}-\Sigma DOF^{f}$$

where $N_{level}^{f}$ is defined as the number of experimental groups at each level of a factor (f). In this experiment, a spcific level of each factor is divided into five groups, namely, $N_{level}^{f}$ = 5 for each factor. *n* is defined as the total amount of data for four types of filling ratios, which is 100 in this experiment.

Then, calculate the sum of the square for each factor and error based on their definitions, given below:

(10) $$SOS^{f}\;=\sum_{i=1}^{5}n_{level}^{f}{\left(K_{i}^{f}-\bar{x}\right)}^{2}$$

(11) $$SOS^{total}=\sum_{i=1}^{100}{\left(x_{i}-\bar{x}\right)}^{2}$$

(12) $$SOS^{error}\;=SOS^{total}-\Sigma SOS^{f}$$

where $n_{level}^{f}$ is defined as the amount of data for four types of filling ratios at each level of a factor (*f*), which is 20 in this experiment. $x_{i}$ represents the experimental results data for four types of filling ratios. $\bar{x}$ represents the average of the experimental data for four types of filling ratios.

Then, we can divide the sum of the square by their degree of freedom to obtain the mean square, which can be donated as $MS^{f}$ for three factors and $MS^{error}$ for the error.

Finally, the F-ratio and the influence contribution percentage of each factor and error are calculated. The F-ratio is used to determine whether a factor affects the result. If it is greater than 1, it has a significant influence. The F-ratio can be computed using the following formula:

(13) $$F^{f}=\frac{MS^{f}}{MS^{error}}$$

The influence contribution percentage is given by the following formula:

(14) $$P=\frac{MS}{MS^{total}}$$

The calculation results are shown in Table 5. The proportions of the influence of the three variables and errors on the experimental results are drawn into a pie chart, as shown in Figure 5d. Among the three variables, the impact of the embossing temperature on the experimental results is the largest, accounting for 48%, while the impact of the embossing time is the smallest, accounting for 17%.

According to the calculation above, the F-ratios of the three factors are all greater than 1, which means that the influences of the three factors on the experimental results are all significant and hence cannot be ignored.

## 4. Discussion

**Figure 6:**
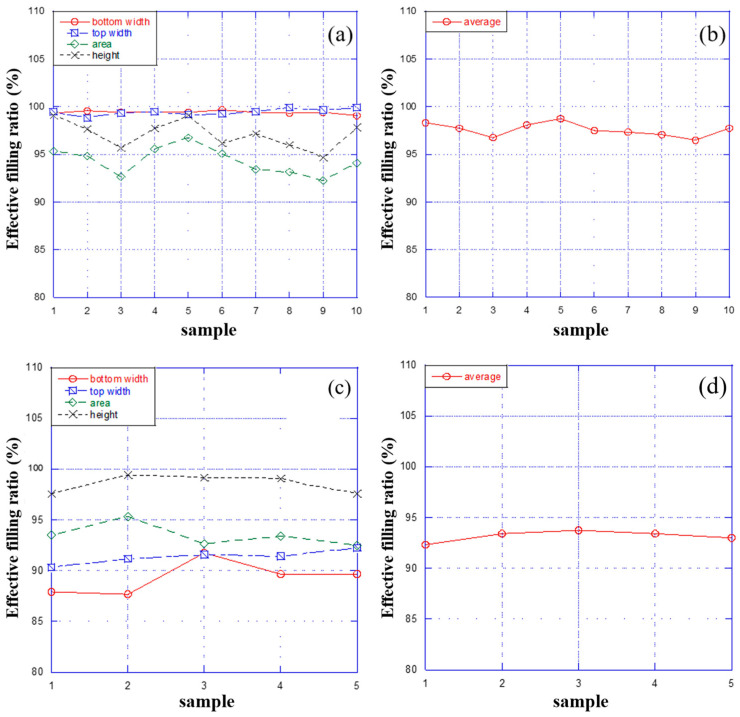
Test results for under the optimal conditions: (**a**) the effective filling ratio for repeating the experiments 10 times; (**b**) the average effective filling ratio for repeating the experiments 10 times; (**c**) the effective filling ratio of mold II for repeating the experiments 5 times; and (**d**) the average effective filling ratio of mold II for repeating the experiments 5 times.

The optimal effective filling ratio for PS polymer is observed by repeating the experiments 10 times under the optimal conditions, which are 130 °C, 400 Pa and 15 min. As shown in Figure 6a, the four effective filling ratios are all at a high level under the optimal conditions, with values from 92% to 100%. After that, the average of the four ratios is calculated, and the curve is drawn in Figure 6b. This indicates that the average effective filling ratio is maintained at a high level of 95% to 100% under such conditions.

The detailed analysis of the embossing force versus the effective filling ratio, as shown in Figure 6a, reveals insights into the performance of microchannel fabrication across different parameters, including the top width, bottom width, area and height. In Figure 6a, the top width (represented by blue squares) and bottom width (represented by red circles) parameters consistently maintain a high effective filling ratio close to 100% across the entire range of embossing forces, indicating precise and stable control during the embossing process. The height parameter (depicted by black crosses) also demonstrates high stability, with an effective filling ratio remaining at around 100% with only minor fluctuations. In contrast, the area parameter (shown by green diamonds) exhibits significant variation, starting at approximately 85–90% at lower forces and fluctuating without stabilizing, suggesting greater sensitivity to changes in embossing force.

To further investigate these findings, the optimal conditions identified in the previous experiment were applied to a different type of master mold (mold II) using the same hot embossing setup, with more details shown in Figure S2 (Supplementary Materials). The results of this experiment, shown in Figure 6c,d, indicate that the effective filling ratios for this new master mold are significantly lower compared to those of the previous mold, ranging between 87% and 100%, with an average between 90% and 95%. This variation underscores that the optimal embossing conditions may not be universally applicable and can vary depending on the type of master mold used. Consequently, while the previous experiment highlighted a consistent performance for width and height parameters, the new findings emphasize the need to adjust the embossing conditions when using different master molds to achieve optimal results.

## 5. Conclusions

Self-made hot embossing equipment is used to investigate the optimum conditions for fabricating microchannels on substrate polymer. The hot embossing can be divided into three processes, and polystyrene (PS) polymer was chosen as the material of substrate polymer in this experiment. To evaluate the microchannels fabricated on substrates more precisely, four effective filling ratios are defined. Then, three main factors are examined using the Taguchi method as the experimental conditions to obtain the results for corresponding filling ratios. Based on that, we found the relationship between the experimental condition and the quality of the fabricated structure on PS polymer. The optimal filling ratio occurs when the temperature, force and holding time are set to 130 °C, 400 kg and 15 min, respectively. By an analysis of variances (ANOVA), we found out that the temperature is the most vital factor in the use of the hot embossing process to fabricate microchannels on the substrate polymer, while embossing force and embossing time are also significant factors. After confirming the optimal experimental conditions, we found that the effective filling ratio is maintained by more than 95%, which is enough for its application in flexible fuel cells. However, when the type of master mold is changed to carry on the same hot embossing process, the optimal conditions turn out to be different. In conclusion, our self-developed hot embossing equipment, optimized using our evaluation approach combined with the Taguchi method, achieves a high effective filling ratio in fabricating columnar structure microchannels on polymer substrates. This capability demonstrates its suitability for producing microchannels tailored for proton-exchange membrane fuel cells (PEMFCs) under ideal process conditions. We expect to further validate the effectiveness of this method through subsequent evaluations and tests of the fuel cells. This method can also be applied to other research areas, such as microchannel fabrication in traditional semiconductor technology and microchannel preparation in biosensors. By utilizing this method to reduce mold size, it is anticipated that nano-scale embossing can be achieved in the future.

## Figures and Tables

**Table 1:** Comparison of different evaluation methods.

Polymer Substrate Used	Configuration of the HE Apparatus for Manufacturing	Functional Parameters	Response Variable	References
PMMA	Traditional-HE	T_e_,P_e_	Bonding quality	[37]
PMMA	Traditional-HE	T_e_,P_e_,t_e_	Relative standard deviation in microchannel width and depth	[38]
PMMA	Traditional-HE	T_e_,Load (kg),t_e_	Microchannel width and depth	[39]
PMMA	Traditional-HE	T_e_,Force (N),t_e_	Bonding quality	[40]

**Table 2:** Levels of embossing temperature, embossing force and embossing time.

Levels	Factors		
	Embossing Temperature (°C)	Embossing Force (kg)	Embossing Time (min)
1	100	100	1
2	110	200	3
3	120	300	5
4	130	400	10
5	140	500	15

**Table 3:** The results of experiments.

Temperature (°C)	Force (kg)	Time (min)	Ratio (%)				
			Bottom Width	Top Width	Area	Height	Average
100	100	1	98.10	99.42	82.01	83.39	90.73
	200	3	99.44	97.80	90.27	90.77	94.57
	300	5	98.10	97.96	91.89	92.07	95.00
	400	10	98.09	95.62	93.20	94.60	95.38
	500	15	98.77	97.69	94.97	99.09	97.63
110	100	3	99.34	94.11	87.48	96.34	94.32
	200	5	98.10	97.29	96.66	96.60	97.16
	300	10	98.21	95.38	94.14	96.38	96.03
	400	15	98.66	98.40	96.77	99.72	98.39
	500	1	97.77	99.56	90.04	91.86	94.81
120	100	5	99.45	94.14	90.39	94.30	94.57
	200	10	98.21	98.79	92.36	97.79	96.79
	300	15	99.56	99.91	99.45	100.70	99.91
	400	1	98.32	99.99	97.47	99.00	98.70
	500	3	98.55	98.97	94.37	98.83	97.68
130	100	10	98.44	98.05	95.89	97.75	97.53
	200	15	98.21	99.56	96.00	98.84	98.15
	300	1	99.01	98.71	96.22	94.15	97.02
	400	3	99.56	99.73	98.69	99.75	99.43
	500	5	98.21	100.00	96.70	100.49	98.85
140	100	15	98.77	97.79	96.27	99.55	98.10
	200	1	98.44	99.05	95.96	99.57	98.25
	300	3	98.32	99.82	95.26	99.57	98.24
	400	5	98.56	98.71	96.50	99.31	98.27
	500	10	99.22	98.45	95.76	98.11	97.89

**Table 4:** The mean value of the average filling ratio for three factors.

Factor (f)	$K_{1}^{f}$	$K_{2}^{f}$	$K_{3}^{f}$	$K_{4}^{f}$	$K_{5}^{f}$	$R^{f}$
Temperature	94.66	96.14	97.53	98.20	98.15	3.54
Force	95.05	96.99	97.24	98.03	97.37	2.98
Time	95.90	96.85	96.77	96.72	98.43	2.53

**Table 5:** The result of the analysis of variances.

Factor	DOF	Sum of Square (SOS)	Mean Square (MS)	F-Ratio (F)	Percent (%)
Temperature (°C)	4	184.76	46.19	5.28	47.51
Force (kg)	4	100.75	25.19	2.89	26.00
Time (min)	4	67.74	16.94	1.95	17.50
Error	87	758.47	8.72	/	8.99
Total	99	1111.72	97.02	/	100

## Data Availability

The data are contained within the article.

## Supplementary Materials

The following supporting information can be downloaded at: https://www.mdpi.com/article/10.3390/mi15081033/s1, Figure S1: Self-made micro hot embossing system: (a) A thermocouple is arranged below the hot embossing platform. (b) Front view of the hot embossing platform (c) The piping layout of the hot embossing platform (d) Complete piping connection; Figure S2: The master mold structure (mold II) used in the experiment.
