# Supplementary material for: Optimization of Hot Embossing Condition Using Taguchi Method and Evaluation of Microchannels for Flexible On-Chip Proton-Exchange Membrane Fuel Cell
Source: Micromachines (Basel). 2024 Aug 14;15(8):1033. doi: 10.3390/mi15081033 (PMC11356460; doi:10.3390/mi15081033)
Supplement: Supplementary file 1 [file micromachines-15-01033-s001.zip › micromachines-3121815-supplementary.pdf]

Supplementary material for

Optimization of hot embossing condition using Taguchi method and evaluation of microchannels for flexible on-chip PEMFC

Yubo Huang [1], Han Gao [1], Zhiheng Wu [2], Hongyang Xiao [2], Cao Xia [1], Yuanlin Xia [1],Zhuqing Wang [1,*]

1 School of Mechanical Engineering, Sichuan University, Chengdu, Sichuan province, 610065, China
2 Sichuan University - Pittsburgh Institute, Sichuan University, Chengdu, Sichuan province, 610225, China
* Correspondence: wzhuqing@scu.edu.cn; Tel.: +86-138-4114-9371

Figure S1 shows more details about our self-made hot embossing equipment.

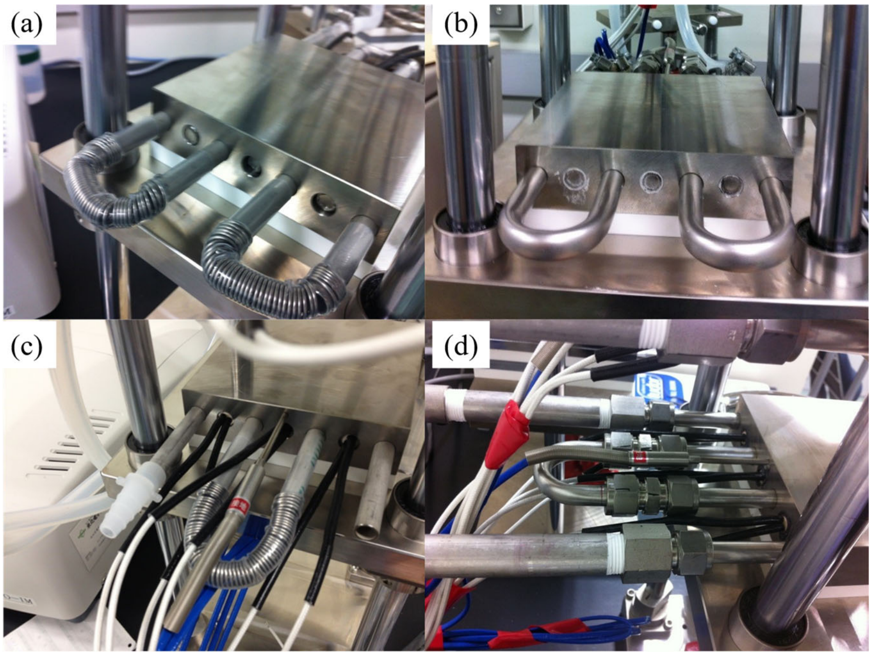

Figure S1 Self-made micro hot embossing system: (a) A thermocouple is arranged below the hot embossing platform. (b) Front view of the hot embossing platform (c) The piping layout of the hot embossing platform (d) Complete piping connection

Figure S2 shows the master mold structure (mold II) used in the experiment.

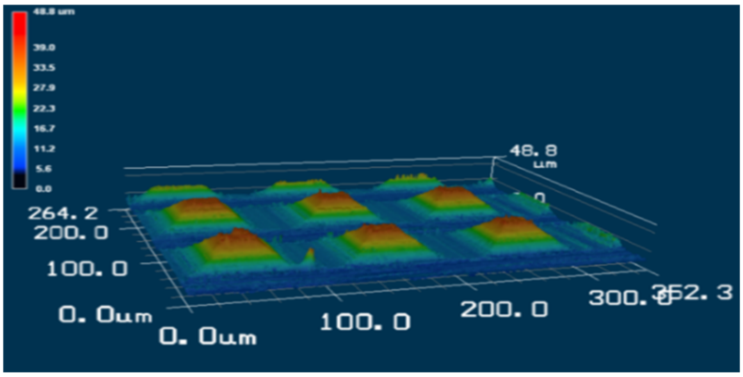

Figure S2 The master mold structure (mold II) used in the experiment
